# Nodule de Sœur Marie-Joseph: pensez à regarder le nombril

**DOI:** 10.11604/pamj.2014.18.289.5036

**Published:** 2014-08-12

**Authors:** Douhi Zakia, Mernissi Fatima Zahra

**Affiliations:** 1 Service de Dermatologie-vénérologie, CHU Hassan II Fès, Maroc

**Keywords:** Nodule, Sœur Marie-Joseph, nombril, tumeur cutanée, Nodule, Sister Marie-Joseph, belly button, skin tumor

## Image en medicine

Nous rapportons le cas d'une jeune patiente de 28 ans, ayant comme antécédent la notion d’épisodes de constipation. Elle a présenté depuis 2 mois une masse d'apparition brutale au niveau de l'ombilic avec notion de suintement. L'examen dermatologique a objectivé une tumeur cutanée et sous cutanée de consistance dure, de couleur rosé, à surface mamelonnée, suintante, ulcérée par endroit, de 1 cm de diamètre et siégeant au niveau de l'ombilic. L’état général était altéré et le reste de l'examen clinique était normal, L’étude histologique a conclu à une localisation ombilicale d'un adénocarcinome bien différencié dont le profil immuno-histochimique oriente vers une origine colorectale (les cellules tumorales expriment la CK20 et CK8/18). L'exploration digestive a confirmée la présence d'une tumeur colique avec une métastase hépatique. Le diagnostic de nodule de Soeur-Marie-Joseph révélant un adénocarcinome du colique métastatique a était retenu; mais l’évolution était fatale. Le nodule de Soeur Marie-Joseph est une métastase nodulaire ombilicale associée à un mauvais pronostic. Il est habituellement révélateur d'un cancer digestif (gastrique ou colique) ou génital. Ce nodule est rare mais caractéristique et mérite d’être connu par les praticiens, car il est facilement accessible à l'examen clinique et sa reconnaissance comme lésion secondaire d'une tumeur solide peut éviter un retard dans la prise en charge de la néoplasie sous-jacente.

**Figure 1:**
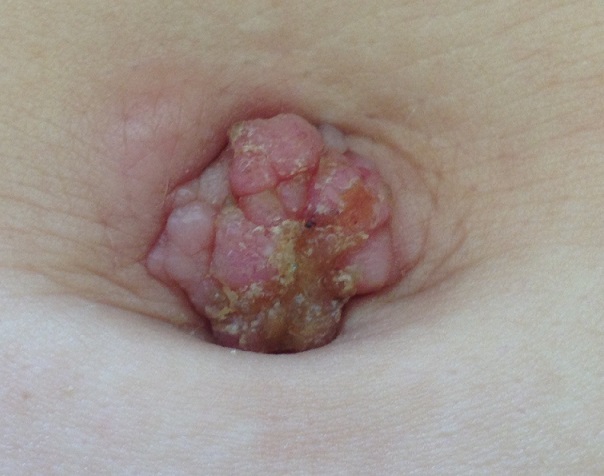
Nodule ombilical érythémateux, de consistance dure, à surface mamelonnée, ulcérée par endroit

